# Determination of radionuclides and elemental composition of clay soils by gamma- and X-ray spectrometry

**DOI:** 10.1186/2193-1801-2-74

**Published:** 2013-02-28

**Authors:** Isinkaye M Omoniyi, Shitta M B Oludare, Oderinde M Oluwaseyi

**Affiliations:** 1 Department of Physics, Ekiti State University, P. M. B. 5363 Ado Ekiti, Nigeria; 2 Department of Physics, University of Ibadan, Ibadan, Nigeria

**Keywords:** Gamma spectrometry, XRF, Clay soil, Enrichment factor, Geoaccumulation index

## Abstract

Radiochemical and elemental analysis of clay soils collected from different locations within Ekiti State have been performed in this study using gamma and XRF spectrometric measurements. The results of this study show that the mean concentrations of uranium ranged from 2.2 ± 1.0 mg/kg to 3.2 ± 1.1 mg/kg, that of thorium ranged from 4.0 ± 0.5 mg/kg to 5.7 ± 1.7 mg/kg, while potasium presented in % by weight ranged from 0.4 ± 0.2 to 1.3 ± 0.3 in all the locations. The overall mean concentrations of these radionuclides are comparable to values from other locations around the world. The XRF analysis revealed 4 major elements and 11 minor or trace elements present in the clay samples. The distribution of the various major and trace elements in all the sampling sites do not follow any systematic trend but vary from point to point. To assess the level of contamination and the possible anthropogenic impact in the clay soils, the enrichment factor (EF) and the geoaccumulation index (Igeo) were estimated for some potential hazardous elements. The results indicate that Cu, Zn, Ni and Mn have EF < 2 indicating minimal or no enrichment while Pb is moderately enriched in all the locations.

## Background

The earth crust contains small amount of uranium, thorium, potassium and other trace and major elements such as Cs, Cd, Pb, Fe, Mg, Mn, etc. The concentrations of all these elements depend on the geology of a local environment as well as other natural and anthropogenic processes. The average concentration of uranium in the earth crust has been reported to be in the range of 2–3 ppm, while thorium exists in the range of 8–12 ppm (IAEA, 2003a). Potassium is widely distributed in nature, with concentrations varying from about 0.1% for limestone, through 1% for sandstones to as much as 3.5% for some granite (Eisenbud, 1987; Eisenbud and Gesell, 1997). The concentrations of major and trace elements in environmental samples had been studied by several authors using either atomic absorption spectrometry (AAS) (Mico et al., 2006; Fagbote and Olanipekun, 2010; Zheng et al., 2010, Ghrefat et al., 2010; Ali and Malik, 2011) or XRF analysis (Kierzek et al., 1999; Boyle, 2000; Baranowski et al., 2002; Zhang et al., 2003; Rauf et al., 2004; Bakraji et al., 2010). Most of these studies indicate high concentrations of major and minor elements in the environment. Pollution of natural environment by metals is a worldwide problem because these metals are indestructible and many of them have toxic effects on living organism, especially when they exceed certain threshold (Forstner, 1990; Ghrefat and Yusuf, 2006).

Soil forms a major component of an ecosystem and is the most endangered due to the influence of various human activities such as urban development, industrial and technological advancements, agricultural practices and indiscriminate waste disposal. Soil is considered contaminated when chemicals are present or other alterations have been made to its natural environment (Gowd et al., 2010). Clay is a natural earthy fine grained inorganic material that develops plasticity when mixed with limited amount of water (McGraw-Hill, 1997; Odo et al. 2008). Its origin could be traced to the breaking down of granite rocks by physical and chemical processes called weathering. Natural clay minerals are well known to mankind from the earliest days of civilization and because of their low cost, abundance in most continents of the world, high sorption characteristics and potential for ion exchange, they form a good material for absorbents (Nayak and Singh, 2007). Clay soils generally contain mostly silica (47%) and alumina (40%), elemental analysis have shown that a great number of minor and trace metallic elements such as Sc, Cr, Cu, Ti, Ga, Zr, Mn, Mg, Sr and Pb exist in clay soil. There are several classes of clay which include; smectites (montmorillonite, saponite), mica (illite), kaolinite, serpentine, pylophyllite (talc), vermiculite and sepiolite (Nayak and Singh, 2007). The specific elemental composition of each clay material will usually depend on the amount of the element present in the host rock, the chemical association of the elements with stable and/or unstable mineral during weathering and the intensity of drainage and other polygenetic alterations associated with clay materials (Ibeanu et al., 1997). It has a wide range of applications in the building and ceramic industries. In the rural area, clay is used for the building of earthen or mud houses, while in the urban area, it is used for making burnt bricks used in building modern dwellings. Clay has been in use for making pottery in different continents of the world for several centuries. It is also used in the manufacturing of refractory’s products and serves as a natural filter for underground water. In some continents of the world, including Africa, it is a common practice for people to engage in the act of eating clay (geophagia) during pregnancy or for curative purposes. All these may lead to direct or indirect accumulation of metals in man.

XRF is a rapid, non-destructive multi-elemental analysis technique with sensitivity in the range of 10^-8^ (IAEA, 2003b) and it is ideal for environmental research. This analytical method has been widely and routinely applied to the analysis of various archaeological samples, historical relics and works of art (Bakraji et al., 2010; Pillay, 2001; Feretti, 2000). XRF can analyze some 15–30 elements with atomic numbers ranging from Z = 11 to Z = 41 and some rare earth elements (REEs) (Bakraji et al., 2010). X-ray fluorescence (XRF) analysis is based on the measurement of characteristics X-rays resulting from de-excitation of inner shell vacancy produced in a sample by means of a suitable source of radiation. Energy-dispersive XRF analysis (EDXRF) employs detectors that directly measure the energy of the X-rays by collecting the ionization products in a suitable detecting medium (Tajani and Markowicz, 2004). Gamma ray spectrometry is another analytical technique used widely in environmental investigations. It is used mainly for the determination of the concentrations of radioactive elements that decay through gamma emission. The use of gamma ray spectrometry as a tool for mapping radioelemental concentrations has found widespread acceptance in diverse fields. The method is widely used for environmental monitoring, geological mapping and mineral exploration. It is also a non-radioanalytical technique. Even though, there are many naturally occurring elements that have radioactive isotopes, only potassium and the uranium and thorium decay series, have radioisotopes that produce gamma rays of sufficient energy and intensity measurable by gamma ray spectrometry due to their relative abundance in nature. Geochemical analysis of the total concentrations of major rock-forming elements is important because they provide valuable information about the geochemical properties of the soil in any given environment. The crossed analysis of geochemical and radiometric data provides a useful tool for a better understanding of the origin and characteristics of different rocks (Brai et al. 2006) and soils. It is reported in literature that higher percentage of some metallic oxides in rocks and soil will result in higher specific activities of primordial radionuclides such as ^238^U, ^232^Th and ^40^ K in such rocks and soil (Brai et al. 2006). The present work is aimed at evaluating the radionuclides and elemental composition of clay soil using gamma- and X-ray spectrometry. It is also the objective of the study to assess the extent and degree of pollution by metals and identify the origin of the metals using the enrichment factor and geo-accumulation index.

## Materials and methods

### Sample collection

The clay samples used for these analyses were collected from five major clay deposits identified within Ekiti State in the south-western part of Nigeria. The study area is situated between longitudes 4^°^ 45˝ to 5^°^ 45˝ East of the Greenwich Meridian and latitudes 7^°^15˝ to 8^°^ 5˝ North of the Equator. The area is completely within the geological basement complex region of Nigeria. In all, 25 samples were analyzed for their radionuclides and elemental concentrations. The samples were collected directly from the exiting mining sites. This gives a good representation of the actual material being utilize either as building material or those used in pottery making. The collected sample were packed into black polythene bags and transported to the laboratory where they were initial air dried at room temperature for about 5 days in order to reduce the moisture content.

### Sample preparation and measurement for XRF analysis

**Figure 1:**
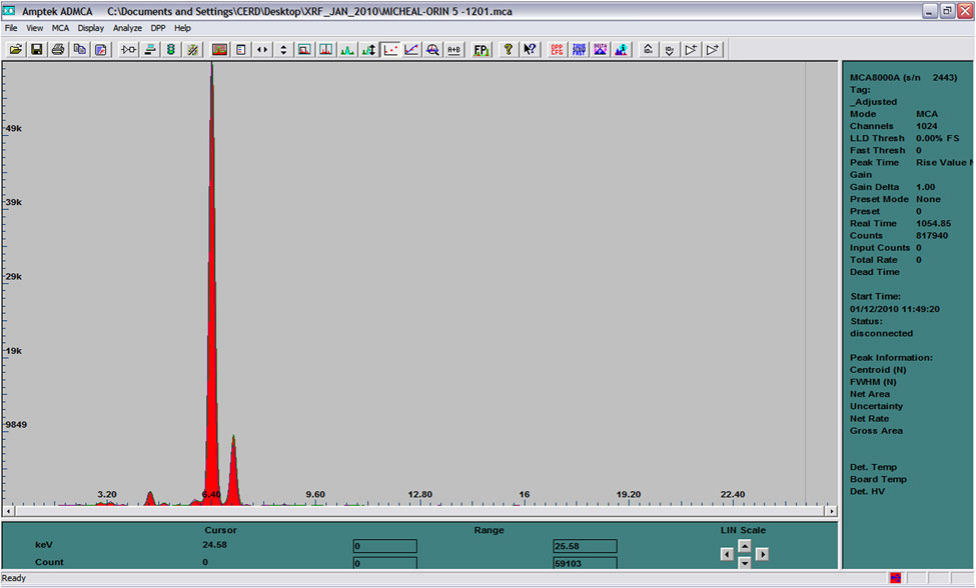
**Linear Scale of Orin-5.** The two major peaks are K- alpha and beta of iron.

**Figure 2:**
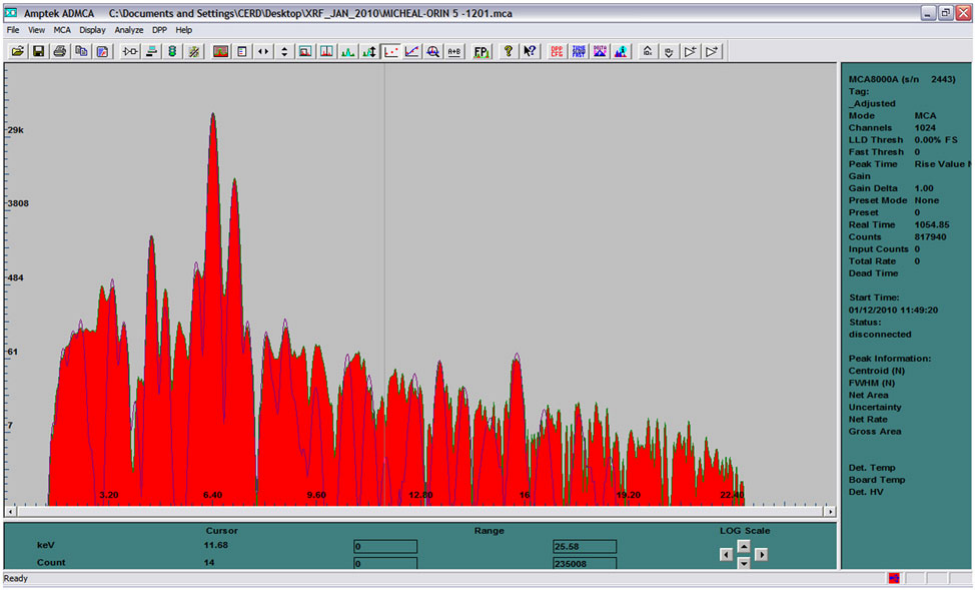
**Logarithm Scale of Orin-5.**

To satisfy homogeneity condition of XRF analysis, the clay samples were pulverized manually to very fine powder with an agate mortar and pestle. Pellets of 13 mm diameter were made from 0.3–0.4 g powder without binder at 8 tons of pressure with a hydraulic press. The pellets were kept in different polythene bags which were in turn kept in polypropylene container until analysis. Each sample pellet was irradiated for 1000 seconds at fixed condition of 25 kV and 50 μA. The elemental analysis of the samples was performed using the Energy Dispersive X-ray Fluorescence (EDXRF) spectrometer at the Centre for Energy Research and Development, Obafemi Awolowo University, Ile-Ife, Nigeria. The EDXRF spectrometer consists of a self-contained miniature X-ray tube system ECLIPSE-III, which includes the X-ray tube with a silver (Ag) transmission target, and a beryllium window, a portable Controller incorporating the power supply and control electronics. The Controller generates all the voltages needed to operate the x-ray tube and provides both voltage (kV) and current (μA) display and control. The X-Ray Detector is a Model XR-100CR, high performance thermoelectrically cooled Si-PIN photodiode, with a preamplifier. The detector is powered by the PX2 CR Power supply, which includes a spectroscopy grade Shaping Amplifier. The detector system has a resolution of 220 eV FWHM, for the 5.9 keV peak of ^55^Fe. The detector is coupled to MCA8000A Multichannel Analyzer for signal processing and data acquisition. The spectrum of Orin sample #5 is shown in Figure 1, while the Logarithm Scale of the same sample is shown in Figure 2. The X-ray tube, ECLIPSE-III with associated Controller/power supply, the Detector system and the Multichannel Analyzer were all supplied by AMPTEK INC., MA USA. The quantitative analysis of the samples was carried out using Fundamental Parameter (FP) method with XRF-FP Software package by CrossRoad Scientific.

### Sample preparation and measurement for Gamma ray spectrometric analysis

Detailed procedure for the preparation and measurement of gamma emitting radionuclides in the clay samples is reported elsewhere (Isinkaye and Shitta, 2010). The air-dried samples were pulverized into powder to achieve uniform matrix similar to the standard sample. All the samples were stored for an upward of 40 d in radon impermeable plastic containers prior to analysis. A 7.6 cm × 7.6 cm NaI(Tl) detector optically coupled to photomultiplier tube was used for the measurement of gamma emitting radionuclides in the clay samples. A multi-channel analyzer matched to an IBM- Compatible personal computer was used for the pulse processing and data analysis. The spectrometer was calibrated against reference material with known activity concentrations of ^226^Ra, ^232^Th and ^40^ K (Isinkaye and Shitta, 2010). The detector has a resolution of 8% at the 0.662 MeV line of ^137^Cs, which is capable of distinguishing the gamma-ray energies of the radionuclides considered in this study. The activity concentrations of ^226^Ra and ^232^Th were determined from the gamma lines associated with their respective short-lived daughters; ^214^Bi (1760 keV) and ^208^Tl (2615 keV). Each of the samples and the background was counted for 10 h. The background spectral was deducted from the gross count to obtain the net count due to the sample alone.

### Evaluation of massic elemental concentrations

The activity concentrations of ^238^U, ^232^Th and ^40^ K in Bqkg^-1^ were converted into massic elemental concentrations in part per million (ppm) units for uranium and thorium, and % by weight for potassium, respectively, according to the following equation (Dragovic et al., 2006):1

where F_E_ is the fraction of element *E* in the sample, *M*_*E*_ is the atomic mass (kg mol^-1^), λ_E,i_ is the decay constant of the measured isotope of element *E* (s^-1^), *f*_*E,i*_ is the fractional atomic abundance in nature, and *A*_*E,i*_ is the measured specific activity (Bq kg^-1^) of the radionuclide under consideration (^238^U, ^232^Th and ^40^ K), *N*_*A*_ is the Avogadro’s number (6.023 × 10^23^ atoms mol^-1^), and *C* is a constant with value of 1,000,000 for U and Th (concentration in ppm) or 100 for K (concentration in % of mass fraction).

## Results and discussion

The concentrations of 15 major and minor elements together with three radionuclides are presented in Tables 1, 2 and 3. The major elements, Fe, Ti, Ca, and K were estimated as their respective oxides and are given in % by weight, while the minor elements, Cu, Zn, Mn, Zr, Ni, Se, Rb, Sr, Nb, Pb and As are presented in mg/kg unit. The naturally occurring radionuclides; U and Th are presented in ppm units while K is presented in % by weight. The discussion on each group is presented below:

**Table 1:** **Mass concentrations of U, Th and K and external gamma dose rate in clay soil samples**

Element	Ado Ekiti	Ara-Ijero Ekiti	Ire Ekiti	Isan Ekiti	Orin Ekiti
	Range	Range	Range	Range	Range
	(Mean ± SD)	(Mean ± SD)	(Mean ± SD)	(Mean ± SD)	(Mean ± SD)
U (ppm)	1.6-4.0	2.0-3.6	1.6-4.1	1.2-3.4	1.5-3.2
	(3.2 ± 1.1)	(2.6 ± 0.7)	(3.1 ± 1.2)	(2.2 ± 1.0)	(2.5 ± 0.8)
^232^Th (ppm)	3.3-7.2	4.2-6.5	2.7-6.4	3.3-4.5	3.7-8.2
	(5.3 ± 1.6)	(5.6 ± 1.0)	(4.2 ± 1.4)	(4.0 ± 0.5)	(5.7 ± 1.7)
K (%)	1.0-1.6	0.1-0.7	0.2-0.9	0.2-0.8	0.3-1.2
	(1.3 ± 0.3)	(0.4 ± 0.3)	(0.5 ± 0.3)	(0.4 ± 0.2)	(0.7 ± 0.3)
Dose rate (nGyh^-1^)	30.0-62.3	26.6-39.5	(22.5-43.7)	(20.6-40.7)	(21.8-49.9)
	(48.1 ± 12.2)	(34.6 ± 10.2)	34.8 ± 6.2	27.2 ± 8.4	37.5 ± 11.3

**Table 2:** **Concentrations of major elements in clay soil samples**

Element	Ado Ekiti	Ara-Ijero Ekiti	Ire Ekiti	Isan Ekiti	Orin Ekiti
	Range	Range	Range	Range	Range
	(Mean ± SD)	(Mean ± SD)	(Mean ± SD)	(Mean ± SD)	(Mean ± SD)
No of Sample	5	5	5	5	5
Fe_2_O_3_ (%)	1.9-17.4	4.9-14.4	3.5-16.4	6.0-22.3	2.8-12.7
	(10.5 ± 7.7)	(11.1 ± 3.9)	(9.7 ± 5.2)	(16.2 ± 6.2)	(7.9 ± 3.9)
TiO_2_ (%)	1.2-4.7	3.6-5.7	0.8-4.2	6.2-10.4	1.3-4.2
	(3.2 ± 1.7)	(4.9 ± 0.9)	(2.8 ± 1.2)	(7.6 ± 1.6)	(2.9 ± 1.4)
CaO (%)	0.9-6.5	1.0-3.5	0.2-2.5	2.3-4.7	0.2-5.0
	(3.9 ± 2.4)	(2.3 ± 1.0)	(1.2 ± 0.9)	(3.3 ± 0.9)	(1.6 ± 1.3)
K_2_O (%)	1.2-9.9	3.8-9.2	0.6-5.8	5.3-9.0	0.7-9.5
	(3.2 ± 1.7)	(5.8 ± 2.0)	(2.8 ± 2.0)	(6.3 ± 1.5)	(4.4 ± 3.3)

**Table 3:** **Concentrations of minor or trace elements in clay soil samples**

Element	Ado Ekiti	Ara-Ijero Ekiti	Ire Ekiti	Isan Ekiti	Orin Ekiti
	Range	Range	Range	Range	Range
	(Mean ± SD)	(Mean ± SD)	(Mean ± SD)	(Mean ± SD)	(Mean ± SD)
Cu (ppm)	32-165	28-190	22-176	18-195	17-174
	(100.6 ± 61.6)	(107.6 ± 63.1)	(91.6 ± 60.8)	143.2 ± 71.9	(82.6 ± 60.9)
Zn (ppm)	90-246	88-290	74-364	95-311	67-246
	(161.0 ± 71.9)	(184.2 ± 76.0)	(202.4 ± 124.3)	257.2 ± 92.5	(166.4 ± 84.7)
Mn (ppm)	219-2823	605-2539	452-3606	780-5296	710-2000
	(1593.0 ± 1190.4)	(1869.4 ± 785.2)	(1682.8 ± 1306.7)	3780.6 ± 1870.7	(1451.4 ± 596.8)
Zr (ppm)	151-953	166-813	46-270	222-486	105-1439
	(443.2 ± 314.5)	(389.0 ± 252.4)	150.2 ± 95.0	332.4 ± 95.7	(439.0 ± 56.4)
Ni (ppm)	88-194	32-269	33-336	27-216	22-273
	(136.8 ± 43.5)	(151.8 ± 95.0)	180.4 ± 128.0	133.2 ± 69.5	(147.2 ± 93.5)
Se (ppm)	23-68	49-97	44-61	43-98	42-101
	(49.0 ± 18.9)	(65.5 ± 21.0)	54.8 ± 8.1	69.5 ± 23.3	(63.3 ± 25.9)
Rb (ppm)	33-104	171-234	30-234	169-282	21-188
	(63.0 ± 31.6)	(202.8 ± 30.4)	107.0 ± 90.4	225.5 ± 50.2	(117.0 ± 74.2)
Sr (ppm)	13-89	91-118	10-90	121-346	23-256
	(57.8 ± 31.9)	(106.8 ± 12.0)	36.6 ± 31.7	225.0 ± 80.4	(91.0 ± 38.8)
Nb (ppm)	35-120	34-94	28-99	54-114	24-79
	(78.3 ± 41.5)	(76.3 ± 28.2)	63.8 ± 29.1	90.5 ± 28.0	(51.8 ± 26.8)
Pb (ppm)	73-499	75-310	72-311	86-317	70-232
	(232.2 ± 164.5)	(199.8 ± 100.9)	174.4 ± 118.0	247.2	(150.6 ± 76.6)
As (ppm)	137-183	ND	ND	ND-49	ND
	(160.0 ± 32.5)			23.0 ± 22.7	

### Naturally occurring radionuclides

The massic elemental concentrations of three naturally occurring radionuclides U, Th and K measured in the clay soil samples investigated in this study are presented in Table 1. Ado Ekiti samples have the highest mean concentration of uranium and potassium, and the highest mean concentration of thorium is found in Orin Ekiti. All the lowest mean concentrations are obtained in Isan Ekiti. The concentrations of the three radionuclides, U, Th and K, ranged from 1.2–4.1 ppm, 2.7–8.2 ppm and 0.1–1.6%, respectively. These radionuclides showed a range of concentrations, as a consequence of varying geological composition of the studied area.

Massic conconcentrations of U, Th and K in comparison with other locations around the world is presented in Table 4. The mean U (ppm) concentration obtained in this study is greater than values obtained in Albenia, Australia, Cyprus and Italy, lower than values obtained in Canada, Egypt and Jordan but comparable to values obtained in Serbia and Montenegro, and USA (Table 4). The mean concentration of Th (ppm) is higher than those obtained in Cyprus and Egypt but lower than those obtained in Albenia, Australia, Bulgaria, Canada, Italy, Jordan, Serbia and Montenegro, and USA. The mean concentration of K (%) is however lower than the values obtained in all these country except Cyprus. All the radionuclides concentrations except uranium are lower than the world average values (Table 4).

**Table 4:** **The comparison of the mass concentrations of U, Th and K in clay soil samples of the present study with other locations around the world**

Location		U (ppm)	Th (ppm)	K (%)	Reference
Albania*		0.48-7.68 (1.84)	0.98-39.5 (5.93)	0.05-3.75 (1.17)	UNSCEAR (2000)
Australia	Rock	2.1-3.6 (2.5)	18-55 (15)	2.4-3.8 (2.4)	Dickson and Scott (1997)
	Soil	1.6-3.8 (2.2)	6-19 (1.3)	0.7-1.9 (1.3)	
Bulgaria*		0.64-15.2 (3.2)	1.73-39.5 (7.41)	0.05-3.75 (1.17)	UNSCEAR (2000)
Canada (Rock)		0.8-16.4 (4.1)	1.1-41.0 (11.9)	1.0-6.2 (3.1)	Killeen (1979)
Cyprus*		0.08-7.2 (1.2)	0.25-13.1 (2.72)	0.04-2.91 (0.58)	Tzortzi et al. (2003)
Italy*		0.16-5.6	0.25-16.8 (5.43)	0.03-5.14 (1.41)	Chiozzi et al. (2002)
Egypt*		0.48-9.6 (2.96)	0.49-23.7 (4.45)	0.09-2.12 (1.04)	UNSCEAR (2000)
Jordan*		1.76-8.32 (6.72)	5.18-25.4 (20.2)	0.45-1.96 (1.82)	Al-Jundi et al. (2003)
Serbia and Montenegro		1.2-6.24 (2.76)	4.45-21.0 (10.4)	0.88-2.99 (1.98)	Dragovic et al. (2006)
USA*		0.32-11.2	0.98-32.1 (8.20)	0.32-2.28 (1.21)	Myrick et al. (1983)
World Average		2.64	11.1	1.37	Dragovic et al. (2006)
Present study		1.2-4.1 (2.7)	2.7-8.2 (4.9)	0.1-1.63 (0.7)	

* Sourced from Dragovic et al. (2006).

### Estimation of gamma dose rate

The radiological implications of the activity concentrations of the naturally occurring radionuclides present in the clay samples were estimated using the external gamma dose rates at 1 m above an infinite homogeneous soil medium per unit radioelement concentration assuming radioactive equilibrium in the uranium and thorium decay series. In the calculation, the contributions of artificial radionuclides such as ^137^Cs and ^90^Sr were neglected. The calculations were performed according to the following equation (IAEA, 2003a; Lovborg, 1984):2

Where, A_K_ is the mass concentration of K in %, A_U_ is the mass concentration of uranium in ppm and A_Th_ is the mass concentration of thorium in ppm. The estimation is based on the assumption that 1%K corresponds to 13.078 nGyh^-1^, 1 ppmU gives 5.675 nGyh^-1^ and 1 ppmTh is equivalent to 2.494 nGyh^-1^dose rate, respectively. The range and mean dose rates obtained for all the clay samples in the study locations are presented in Table 1. All the mean gamma dose rates obtained for the five study locations are lower than the world average value of 59 nGyh^-1^ (UNSCEAR, 2000). The effective dose was also estimated using the formula suggested by Dragovic et al., (2006):3

Where D is the gamma dose rate obtained from mass concentrations of U, Th and K, 0.7 SvGy^-1^ is the conversion coefficient from absorbed dose in air to effective dose and 0.2 represents the outdoor occupancy factor, which shows that the people in the study area spend ~20% of their time outdoor. The mean annual effective dose obtained in the study area varied from 0.03–0.06 mSv., which fall below the worldwide mean annual effective dose value of 0.07 mSv. The results obtained indicate that the study area can be categorized as area with normal background radiation.

### Major elements

The lowest mean concentration value (7.9 ± 3.9%) of Fe_2_O_3_ obtained in this study is found in Orin-Ekiti while the highest mean concentration value of 16.2 ± 6.2% is obtained in Isan-Ekiti. The values obtained in all sampling points ranged from 1.9–22.3%. The mean concentrations of Fe_2_O_3_ in all the five locations are higher than the average crustal value reported in Turekian and Wedepohl (1961). The values are also higher than mean value obtained in a Mediterranean agricultural soil in Spain. The high concentration of Fe in the clay soils is generally not of any major concern because Fe is not a contaminant element. Fe is important in plant nutrition and an essential crop micronutrient. The mean concentrations of TiO_2_ follow the same trend as Fe_2_O_3_ with lowest mean value (2.9 ± 1.4%) obtained in Orin-Ekiti while the highest mean value of 7.9 ± 1.6% is obtained in Isan-Ekiti. TiO_2_ is the most common compound of Titanium and is widely distributed in the Earth’s crust. It is found in almost all living things, rocks, water bodies and soil (Wikipedia, 2011). Its proportion in soil is approximately 0.5–1.5% (Barksdale, 1968). The concentration of CaO varied from 0.2–6.5% with highest mean concentration found in Ado-Ekiti and the lowest concentration obtained in Ire-Ekiti. Similarly, the concentrations of K_2_O vary from 0.6–9.9% with highest and lowest mean concentrations of 6.3 ± 1.5% and 2.8 ± 2.0% respectively. The Ca and K concentrations obtained in this study are comparable to the values obtained by XRF analysis of some clay samples in Pakistan (Baranowski et al., 2002). Their results indicate a range of Ca to be 0.10–8.9% while K ranged from 0.05–2.25%. Also the levels of Ca and K obtained in this study is higher than mean concentrations of 0.35% and 0.24% obtained respectively for Ca and K in coal samples by Kierzek et al. (1999).

### Minor elements

The results showed that Mn has the highest overall mean concentration, followed by Zr, Pb, Zn, Sr, Nb, Se, As (Table 3). All the sampling locations showed higher Cu, Zn, Mn, Ni, Pb and As contents than the values obtained for average shale as reported by Turekian and Wedepohl (1961). As seen, the distributions of these minor elements vary much from different sampling locations i.e the distribution does not follow any systematic trend. For Cu, the mean concentrations vary from 82.6 ± 60.9-143.2 ± 71.9 mg/kg, Zn, 166.4 ± 84.7-257.2 ± 92.5 mg/kg, Mn, 1451.4 ± 596.8-3780.6 ± 1870.7 mg/kg, Zr, 150.2 ± 95.0-443.2 ± 314.5 mg/kg, Ni, 133.2 ± 69.5-180.4 ± 128.0 mg/kg, Se, 49.0 ± 18.9-69.5 ± 23.3 mg/kg, Rb, 63.0 ± 31.6-225.5 ± 50.2 mg/kg, Sr, 36.6 ± 31.7-225.0 ± 80.4 mg/kg, Nb, 51.8 ± 26.8-90.5 ± 28.0 mg/kg, Pb, 150.6 ± 76.6-247.2 ± mg/kg, and As, 23.0 ± 22.7-160.0 ± 32.5 mg/kg. The high standard deviation values indicate the spread in the distribution of the minor elements in all the sampling sites. Most of the minor elements have their highest mean concentrations at Isan-Ekiti sampling sites. Isan-Ekiti clay is kaolinitic in nature and it is used extensively in making earthen vessels used by local populace for cooking. This could pose metal poisoning and some other detrimental health hazard. Some of the potentially hazardous element such as Cu, Zn, Ni, Pb and As have their mean concentrations higher than the maximum allowable concentration values in clay soil as applied in the Federal Republic of Germany. For Cu, Zn, Ni and Pb, the maximum allowable concentration in clay soil are respectively, 60 mg/kg, 200 mg/kg, 70 mg/kg and 100 mg/kg.

### Enrichment factor and geoaccumulation index

In order to assess the level of contamination and the possible anthropogenic impact in the clay samples, the enrichment factors (EF) and geoaccumulation index (I_geo_) were estimated for some selected potentially hazardous elements evaluated in this study. The enrichment factor is defined as:4

Where C_x_ is the concentration of the potentially enrichment element and C_Fe_ is the concentration of the proxy or normalizing element usually Fe. The world average elemental concentrations reported by Turekian and Wedepohl (1961) in the earth’s crust were used as reference in this study because regional geochemical background values for these elements are not available. Five major contamination categories are recognize on the basis of the enrichment factor, where, *EF* < 2 is deficient to minimal enrichment, 2 ≤ *EF* < 5 is moderate enrichment,5 ≤ *EF* < 20 is significant enrichment, 20 ≤ *EF* < 40 means high enrichment, and *EF* > 40 indicates extremely high enrichment. EF can easily be used to differentiate between elemental concentrations from anthropogenic source and those from natural origin. According to Zhang and Liu (2002), EF values between 0.5 and 1.5 indicate the metal is entirely from crustal materials or natural origin, while *EF* > 1.5 suggests that the sources are more likely to be anthropogenic (Ghrefat et al., 2010). The results of the present study show EF values of Cu, Zn, Ni and Mn which are all < 2(Table 5), indicating no or minimal enrichment. Pb is moderately enriched in all the locations while As is moderately enriched only in Ado-Ekiti clay samples. All the potentially hazardous elements considered in the study originate from the source rock except Pb which has *EF* > 1.5, indicating anthropogenic source.

**Table 5:** **Average concentrations of selected elements, average shale (ppm) (Turekian and Wedepohl,**^1961^**), enrichment factor and the geoaccumulation index values in the clay samples**

Elements	Average Shale Value	Ado Ekiti	EF	I_geo_	Ara-Ijero Ekiti	EF	I_geo_	Ire Ekiti	EF	I_geo_	Isan Ekiti	EF	I_geo_	Orin Ekiti	EF	I_geo_
**Cu**	45	100.6	1.00	0.58	107.6	1.01	0.67	91.6	0.99	0.44	143.2	0.92	1.09	82.6	1.09	0.29
**Zn**	95	161	0.76	0.18	184.2	0.82	0.37	202.4	1.03	0.51	257.2	0.79	0.85	166.4	1.04	0.22
**Ni**	68	136.8	0.90	0.42	151.8	0.95	0.57	180.4	1.29	0.82	133.2	0.57	0.39	147.2	1.29	0.53
**Mn**	850	1593	0.84	0.32	1869.4	0.93	0.55	1682.8	0.96	0.40	3780.6	1.29	1.57	1451.4	1.02	0.19
**Pb**	20	232.2	5.20	2.95	199.8	4.23	2.74	174.4	4.23	2.54	247.2	3.59	3.04	150.6	4.48	2.33
**As**	13	160	5.51	3.04	-	-	-	-	-	-	23	0.51	-	-	-	-

The geo-accumulation index (Igeo) originally introduced and applied by Muller (1969) was used to evaluate the degree of elemental pollution in the clay soils from the study area. Mathematically, Igeo is given as (Zheng et al., 2010; Matini et al., 2011):5

Where C_n_ is the concentration of the potentially hazardous trace element (e.g Cu, Ni, Pb, etc) in the clay sample, B_n_ is the geochemical background value in average shale (Turekian and Wedepohl, 1961) of element n and *k* = 1.5 is the background matrix correction factor introduced to account for possible differences in the background values due to lithogenic effects. The results of the geo-accumulation index obtained in this study indicate that Cu is moderately contaminated in Isan Ekiti clay with *I*_*geo*_ = 1.09(Tables 5 and 6). Pb is moderately/ strongly contaminated in all the sampled locations. The anthroponenic sources of Pb include; exhaust fumes from motor-vehicle, smelting activities, indiscriminate dumping of used lead acid batteries, etc.

**Table 6:** **Geoaccumulation index (I**_**geo**_**) for contamination levels in clay soil samples**

I_geo_class	I_geo_value	Contamination level
0	*I*_*geo*_ ≤ 0	Uncontaminated
1	0 < *I*_*geo*_ ≤ 1	Uncontaminated/moderately contaminated
2	1 < *I*_*geo*_ ≤ 2	Moderately contaminated
3	2 < *I*_*geo*_ ≤ 3	Moderately/strongly contaminated
4	3 < *I*_*geo*_ ≤ 4	Strongly contaminated
5	4 < *I*_*geo*_ ≤ 5	Strongly/extremely contaminated
6	*Igeo* > 5	Extremely contaminated

### Statistical analysis

**Figure 3:**
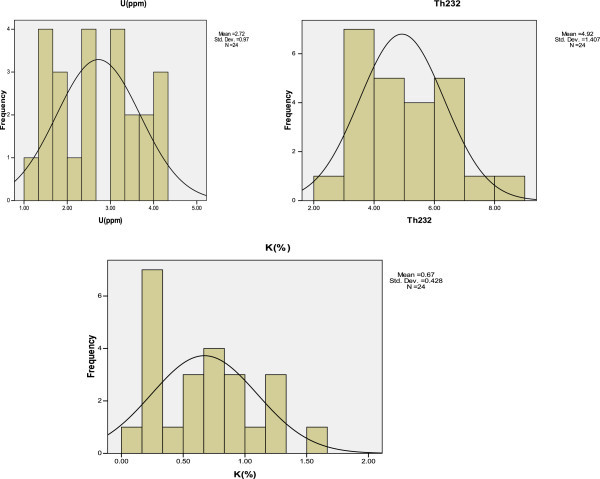
**Frequency distribution of the mass concentrations of U, Th and K in clay soil.**

Table 7 gives the descriptive statistics for the massic elemental concentrations of U, Th and K for all the measured clay samples. These includes; arithmetic means, median, standard deviation, maximum, minimum, skewness and Kurtosis, while the frequency distributions are presented in Figure 3.

**Table 7:** **Descriptive statistics of massic elemental concentrations of U, Th and K for all the analyzed clay samples**

Parameter	Massic elemental concentration		
	U (ppm)	Th (ppm)	K (%)
Mean	2.7212	4.9229	0.6716
Standard deviation	0.96973	1.40750	0.42847
Maximum	4.11	8.20	1.63
Minimum	1.17	2.73	0.09
Median	2.6215	4.5394	0.6670
Skewness	−0.005	0.564	0.518
Kurtosis	−1.366	−0.384	−0.596

The results of the Pearson correlation coefficients between the naturally occurring radionuclides and the major and trace elements are presented in Table 8. The results indicate a strong positive correlation between radioactive K and As. This radioactive K has a poor negative correlation with almost all the other elements except Ca, Se, Nb and Pb. U correlates significantly only with Th and Sr at 95% confidence level. Th does not interact significantly with any of the major and minor elements. Fe correlates significantly with Ti, Cu, Zn, Mn, Ni, Se, Rb, Nb and Pb, whereas Ti has strong interactions with Cu and Mn. There exists a strong correlation between Ca and As; K and Sr; Cu and Zn, Mn, Ni, Se, Rb, Nb, Pb; Zn and Mn, Ni, Se, Rb, Nb, Pb; Mn and Se, Rb, Nb, Pb. Ni correlates strongly with Se, Rb, Nb, Pb while Se has a strong correlates with Rb, Nb,Pb. Strong interactions also exist between Pb and As. All the three radionuclides considered in this study are poorly correlated with the measured major and trace elements indicating different geochemical behavior. Positive correlations however exist between most of the elemental pairs in the clay samples suggesting the same source or co-contamination. Negative or inverse correlations between variables indicate that the variable pairs are derived from different origin and that such do not associate in their geochemical dynamics.

**Table 8:** **Pearson correlation coefficients between natural radionuclides and elemental concentrations in clay soil samples**

Variable	K(%)	U(ppm)	Th(ppm)	Fe	Ti	Ca	K	Cu	Zn	Mn	Zr	Ni	Se	Rb	Sr	Nb	Pb	As
K(%)	1																	
U(ppm)	.499^*^	1																
Th(ppm)	.299	.426^*^	1															
Fe	-.022	.079	.161	1														
Ti	-.292	-.296	.001	.574^**^	1													
Ca	.170	-.194	-.019	.406^*^	.215	1												
K	-.472^*^	-.366	.103	.157	.244	.486^*^	1											
Cu	-.020	.030	.173	.910^**^	.592^**^	.229	-.007	1										
Zn	-.165	-.075	.105	.864^**^	.499^*^	.113	.001	.906^**^	1									
Mn	-.176	-.071	.014	.913^**^	.577^**^	.272	.184	.866^**^	.858^**^	1								
Zr	-.100	-.085	.015	-.198	-.096	.406^*^	.389	-.263	-.292	-.139	1							
Ni	-.056	.074	.291	.550^**^	.239	-.172	-.264	.762^**^	.775^**^	.501^*^	-.369	1						
Se	.121	.186	.342	.590^**^	.153	-.140	-.116	.639^**^	.651^**^	.616^**^	-.496^*^	.534^**^	1					
Rb	-.252	-.113	.084	.765^**^	.446^*^	-.016	.104	.758^**^	.823^**^	.796^**^	-.305	.546^**^	.753^**^	1				
Sr	-.446^*^	-.452^*^	-.194	.216	.454^*^	.503^*^	.695^**^	.062	.077	.317	.417^*^	-.258	-.149	.165	1			
Nb	.065	.059	.139	.806^**^	.328	.204	-.192	.835^**^	.827^**^	.779^**^	-.297	.677^**^	.671^**^	.717^**^	-.104	1		
Pb	.161	.248	.383	.839^**^	.397^*^	.333	-.049	.843^**^	.783^**^	.741^**^	-.149	.678^**^	.622^**^	.600^**^	.031	.816^**^	1	
As	.570^**^	.224	.147	.414^*^	.067	.591^**^	-.310	.343	.215	.223	.075	.145	.062	-.016	-.099	.430^*^	.564^**^	1

*Significant at the 0.05 level.

** Significant at the 0.01 level.

## Conclusion

Radiometric and elemental investigations performed on clay soils reveal the presence of three naturally occurring radionuclides and fifteen major and trace elements. The results show that the radionuclides and elemental concentrations varied widely among the sampling locations. Most of the elements have higher concentrations than the baseline values. The concentrations of U (ppm), Th (ppm) and K (%) are comparable with results from other locations around the world and lower than the world average except U. Soil pollution in the present study was assessed using enrichment factor and geoaccumulation index values. The results indicate that the clay soil samples examined in this study are unpolluted with Cu, Zn, Ni, and Mn. Pb is moderately contaminated in all the sampling locations. The study shows that all the potentially hazardous elements originate from the source rock except Pb, which has EF > 1.5, indicating anthropogenic source.

Descriptive statistics and correlation analysis was carried out on the results in order to have a better understanding of the complex dynamics of the measured parameters. The Pearson correlation analysis shows poor interactions between radionuclides and elemental concentrations. Strong positive correlations were observed among most elemental pairs suggesting the same origin and similar geochemical behavior.

## References

[CR1_141] Ali SM Malik RN Spatial distribution of metals in top soils of Islamabad City, Pakistan Environ Monit Assess 2011 172 1 16 10.1007/s10661-010-1314-x 20140510

[CR2_141] Al-Jundi J Al-Bataina BA Abu-Rukah Y Shchadch HM Natural radioactivity concentrations in soil samples along the Amman Aqaba Highway, Jordan Radiat Meas 2003 36 555 560 10.1016/S1350-4487(03)00202-6

[CR3_141] Bakraji EH Itlas M Abdulrahman A Issa H Abboud R X-ray fluorescence analysis for the study of fragments pottery excavated at Tell Jendares site, Syria, employing multivariate statistical analysis J Radioanal Nucl Chem 2010 285 455 460 10.1007/s10967-010-0595-4

[CR4_141] Baranowski R Rybak A Baranowska I Speciation Analysis of Elements in Soil Samples by XRF Polish J Environ Stud 2002 11 5 473 482

[CR5_141] Barksdale J Hampel CA "Titanium" The Encyclopedia of the Chemical Elements 1968 New York Reinhold Book Corporation 732 738

[CR6_141] Boyle JF Rapid elemental analysis of sediment samples by isotope source XRF J Paleol 2000 23 213 221 10.1023/A:1008053503694

[CR7_141] Brai M Belli S Hauser S Puccio P Rizzo S Basile S Marrale M Correlation of radioactivity measurements, air kerma rates and geological features of Sicily Radiat Meas 2006 41 461 470 10.1016/j.radmeas.2005.09.004

[CR8_141] Chiozzi P Pascale V Verdoya M Naturally occurring radioactivity at the Alps-Apennines transition Radiat Meas 2002 35 147 154 10.1016/S1350-4487(01)00288-8

[CR9_141] Dickson BL Scott KM Interpretation of aerial gamma ray surveys-adding the geochemical factors AGSO J Australia Geol Geophys 1997 17 2 187 200

[CR10_141] Dragovic S Lj J Onjia A Bacic G Distribution of primordial radionuclides in surface soils from Serbia and Montenegro Radiat Meas 2006 41 611 616 10.1016/j.radmeas.2006.03.007

[CR11_141] Eisenbud M Environmental radioactivity 1987 Orlando, USA Academic Press

[CR12_141] Eisenbud M Gesell T Environmental radioactivity from natural, industrial and military sources 1997 San Diego, California, USA Academic Press

[CR13_141] Fagbote EO Olanipekun EO Evaluation of the status of heavy metal pollution of soil and plant (Chromolaena odoranta) of Agbabu bitumen deposit area, Nigeria American-Eurosian J Sci Res 2010 5 4 241 248

[CR14_141] Feretti M Creagh DC Bradley DA Radiation in art and archaeometry 2000 Amsterdam Elsevier 285

[CR15_141] Forstner U Contaminated sediments. Lecture Notes in Earth Science, vol 21 1990 Berlin Springer-Verlag

[CR16_141] Ghrefat HA Yusuf N Assessment Mn, Fe, Cu, Zn, and Cd pollution in bottom sediments of Wadi Al-Arab Dam. Jordan Chemosphere 2006 65 2114 2121 10.1016/j.chemosphere.2006.06.043 16875712

[CR17_141] Ghrefat HA Abu-Rukah Y Rosen MA Application of geoaccumulation index and enrichment factor for assessing metal contamination in the sediments of Kafrain Dam, Jordan Environ Monit Assess 2010 10.1007/s10661-010-1675-1 20839049

[CR18_141] Gowd SS Reddy M Govil PK Assessment of heavy metal contamination in soils at Jajmau (Kanpur) and Unnao industrial areas of the Ganga Plain, Uttar Pradesh, India J Hazard Mat 2010 174 113 121 10.1016/j.jhazmat.2009.09.024 19837511

[CR19_141] International Atomic Energy Agency. Guidelines for radioelement mapping using gamma ray spectrometry data. IAEA-TECDOC-1363 2003a Vienna, Austria IAEA

[CR20_141] International Atomic Energy Agency. Collection and preparation of bottom sediment samples for analysis of radionuclides and trace elements. IAEA-TECDOC-1360 2003b Vienna, Austria IAEA

[CR21_141] Ibeanu IGE Dim LA Mallam SP Akpa TC Munithya J Non-Destructive XRF Analysis of Nigerian and Kenyan Clays J Radioanal Nucl Chem 1997 221 1–2 207 209 10.1007/BF02035268

[CR22_141] Isinkaye MO Shitta MBO Natural radionuclide content and radiological assessment of clay soils collected from different sites in Ekiti State, southwestern Nigeria Radiat Prot Dosim 2010 139 4 590 596 10.1093/rpd/ncp284 20042431

[CR23_141] Kierzek J Malozewska-Bucko B Bukowski P Parus JL Ciurapinski A Zaras S Kunach B Wiland K Assessment of coal and ash environmental impact with the use of gamma- and X-ray spectrometry J Radioanal Nucl Chem 1999 240 1 39 45 10.1007/BF02349134

[CR24_141] Killeen PG Gamma ray spectrometric methods in uranium exploration – Application and interpretation. In: Geophysics and Geochemistry in the Search for Metallic Ores, edited by PJ Hood Geophysical Survey of Canada Economic Geology Report 1979 31 163 230

[CR25_141] Lovborg L The calibration of portable and airborne gamma ray spectrometers- theory, problems and facilities 1984 Roskilde Report Riso-M-2456

[CR26_141] Matini L Ongoka PR Tathy JP Heavy metals in soil on spoil heap of an abandoned lead ore treatment plant, SE Congo-Brazzaville African J Environ Sci Tech 2011 5 2 89 97

[CR27_141] Encyclopedia of Science and Technology; 15th edn vol 3 1997 New York McGraw-Hill Book Company

[CR28_141] Mico C Peris M Sanchez J Recatala L Heavy metal content of agricultural soils in a Mediterranean semiarid area: the Segural River valley (Alicante, Spain) Spanish J Agric Res 2006 4 4 363 372

[CR29_141] Muller G Index of geoaccumulation in sediments of the Rhine River Geol J 1969 2 109 118

[CR30_141] Myrick TE Berven BA Haywood FF Determination of concentration of selected radionuclides in surface soil in the U.S Health Phys 1983 45 631 642 10.1097/00004032-198309000-00006 6885472

[CR31_141] Nayak PS Singh BK Instrumental characterization of clay by XRF, XRD and FTIR Bulletin Mat Sci 2007 30 3 235 238 10.1007/s12034-007-0042-5

[CR32_141] Odo JU Mba AC Udenya TC Effect of agricultural waste ash additives on refractory properties of a blend of two Nigerian clays J Metallurgy Mat Eng 2008 3 1 30 34

[CR33_141] Pillay AE Analysis of archaeological artefacts: PIXE, XRF or ICP-MS? J Radioanal Nucl Chem 2001 247 3 593 595 10.1023/A:1010607332557

[CR34_141] Rauf MA Ikram M Iqbal MJ Manzoor S Comparizon of catalytic activity of clays on locally availablepetroleum fractions J Chem Soc Pakistan 2004 26 1 10 13

[CR35_141] Tajani A Markowicz A EDXRF analysis of thin samples IAEA-TECDOC-1401. Quantifying uncertainty in nuclear analytical measurements 2004 Vienna, Austria International Atomic Energy Agency (IAEA)

[CR36_141] Turekian YY Wedepohl KH Distribution of the elements in some major units of the earth’s crust Geol Soc America 1961 72 175 192 10.1130/0016-7606(1961)72[175:DOTEIS]2.0.CO;2

[CR37_141] Tzortzi M Tsertos H Christisfides S Christodoulides G Gamma-ray measurements of naturally occurring radioactive samples from Cyrus characteristic geological rocks Radiat Meas 2003 37 221 229 10.1016/S1350-4487(03)00028-3

[CR38_141] United Nations Scientific Committee on Effects of Atomic Radiation (UNSCEAR), Sources and Effects of Ionizing Radiation 2000 New York United Nations

[CR39_141] The Free Encyclopedia 2011

[CR40_141] Zhang J Liu CL Riverine composition and estuarine geochemistry of particulate metals in China-Weathering features, anthropogenic impact and chemical fluxes Estuarine Coastal Shelf S 2002 54 1051 1070 10.1006/ecss.2001.0879

[CR41_141] Zhang Y Erhkang L Deyi L Yinsong W Yuehchang Y Changwan W Waiguo S Min Z Guilin Z Yan L PIXE and radioactivity measurements for elemental determination in river water and sediment samples J Radioanal Nucl Chem 2003 258 2 415 419 10.1023/A:1026214627426

[CR42_141] Zheng LG Liu GJ Kang Y Yang RK Some potential hazardous trace elements contamination and their ecological risk in sediments of western Chaohu Lake, China Environ Monit Assess 2010 166 379 386 10.1007/s10661-009-1009-3 19484367

